# Investigation of Leptospirosis Agents in Cattle and Humans

**DOI:** 10.21315/mjms2024.31.5.11

**Published:** 2024-10-08

**Authors:** Perihan Şerifoğlu Bağatir, Osman Aktaş

**Affiliations:** 1 Veterinary Control Institute Directorate, Erzurum, Türkiye; 2 Atatürk University, Faculty of Medicine, Department of Medical Microbiology, Erzurum, Türkiye

**Keywords:** Leptospirosis, ELISA, microagglutination test, real-time PCR, Türkiye

## Abstract

**Background:**

Leptospirosis, a global zoonotic disease, causes serious morbidity and mortality generally in low-income societies. This study aimed to investigate the prevalence of Leptospira serovars in cattle and high-risk human populations.

**Methods:**

This study investigated the presence of pathogenic *Leptospira* serogroups in the blood and kidney samples of cattle arriving at the Erzurum Meat and Milk Institution for slaughter between April and July, and between September and December 2022, and in the serum samples of humans. Kidney and serum samples from 289 cattle and serum samples from 100 individuals from at-risk occupational groups (58 farmers, 25 veterinarians and 17 butchers) were collected. As a control, 100 human blood samples were collected from civil servants who had no contact with animals. Microagglutination testing was used to investigate *Leptospira* serogroups in bovine sera, real-time polymerase chain reaction (PCR) for *Leptospira* DNA in kidney samples, and microagglutination testing and enzyme-linked immunosorbent assays for *Leptospira* antibodies in human blood serum samples.

**Results:**

Microagglutination test in 4.5% of cattle; *Leptospira* DNA was positive in 0.7%. Six strains of *Leptospira interrogans*, two of Bratislava, one of Pomana and one of Icterohaemorrhagiae were found in cattle, as well as one strain of *Leptospira kirschneri* Dadas. In humans, two Icterohaemorrhagiae, one Hebdomadis and one Dadas serovar were detected in both the risk group and the control group. Using ELISA, antibody positivity was found to be 14.0% in the risk group and 11.0% in the control group.

**Conclusion:**

The seroprevalence of *Leptospira spp*. in cattle in Erzurum, Türkiye, is relatively high. In this region, the risk of encountering *Leptospira* in the normal population is as high as in the risk group.

## Introduction

*Leptospira* spirochetes cause Weil’s disease, an infectious disease also known as leptospirosis, swineherd’s disease, Weil-Vasiliev disease, rice field fever, waterborne fever, nanukayami fever, cane fever, swamp fever, mud fever, Stuttgart disease and canicola fever (1). This disease, which causes fatal complications in humans, also causes stillbirths, miscarriages, infertility, weak calf births, and decreased milk production and yield in cattle (2). It is the most important zoonotic bacterial disease worldwide, often affecting resource-limited populations with significant morbidity and mortality. Leptospirosis, whose effects range from a self-limiting systemic infection to a fatal disease accompanied by multiple organ failure, is increasing in incidence worldwide, especially in tropical regions, and it is estimated to cause approximately 60,000 deaths annually (3, 4).

*Leptospira* bacteria spread to rural and urban environments through the urine of chronically infected animals that do not show symptoms of leptospirosis (5). Contaminated environments containing large numbers of live bacteria are the most important sources of infection with *Leptospira*. Leptospirosis is known to be associated with increased soil moisture, and more than 97% of leptospirosis-positive cases occur during the harvest season between August and October, with farmers most commonly affected (6). The risk of contracting this disease is high among veterinarians, butchers and hunters through occupational exposure as well as among those living in low socioeconomic conditions, such as in poverty and with underdeveloped sewage networks and inadequate water supplies (7).

The clinical signs and symptoms of leptospirosis overlap with those of other infections, making diagnosis challenging. This may cause delays in treatment and difficulties in determining the true incidence of the disease. The true status of leptospirosis requires collaboration between veterinary and medical researchers (8). Therefore, differential diagnosis methods, such as microscopic agglutination tests (MAT), indirect hemagglutination, immunoenzymatic assays (ELISA) and culturing from urine or tissues, are used to detect specific antibodies, and so dark field microscopy, immunostaining or polymerase chain reaction (PCR) are needed (9, 10).

In Turkey, there has been no research on *Leptospira* using the PCR method in cattle kidneys. However, using the PCR method, pathogenic *Leptospira* was reportedly found in the urine of 9.4% of cattle slaughtered in Diyarbakır and 4.9% of cattle slaughtered in Elazığ (11, 12). The limited number of studies on leptospirosis in the Erzurum region and the desire to contribute to the literature on *Leptospira* epidemiology played an important role in us initiating this study. The main purpose of our study is to determine the leptospirosis seroprevalence in a risk group consisting of livestock breeders, butchers, and veterinarians in Erzurum and in the normal population, and to detect the differences between the seroprevalences of the two groups. In addition, this study aims to investigate the presence of *Leptospira* in the kidney samples of cattle using molecular methods and to compare them with serological results.

## Methods

### Samples and Determination of Animal Sample Size

In our prospective study, which was conducted during the spring and autumn of 2022, 289 bovine blood serum and kidney samples and 200 human blood serum samples were tested for *Leptospira spp*. using PCR test and MAT. People under the age of 18 years old and cattle under the age of 1 year old were not included in the study. Data from the studies were exported to Microsoft Excel for Mac (version 16.70) for statistical analysis and tabulation purposes. This programme was also used to calculate the size of the cattle samples. A study by Temur and Sağlam in the Erzurum region reported a 24.2% positivity rate, which was accepted as the estimated prevalence (13). The sample size was calculated to be at least 282 samples in total, with a 95% confidence interval, a 5% margin of error and an estimated prevalence of 24.2%.

### Collection and Storage of Clinical Samples

Blood and kidney samples were taken from 289 cattle that came to the Erzurum Meat and Milk Institution for slaughter from Erzurum province and its districts between April and July, and between September and December 2022. Blood samples of at least 5 mL were taken from the carotid arteries of the animals before slaughter, in tubes that did not contain anticoagulants. Kidney samples taken after slaughter were placed in sterile sample bags and brought to the laboratory in a cold chain. The sera obtained from the blood samples in the laboratory were transferred into Eppendorf tubes and stored at −20 °C until the MAT was performed. The kidney samples were transferred to 2 mL sterile screw-cap bead tubes and phosphate-buffered saline (pH 7.4) was added. The tubes were kept in the freezer at −20 °C until the extraction time. Blood samples were taken from 100 people at risk of leptospirosis (animal owners, butchers and veterinarians) and from 100 civil servants who had no contact with animals as a control group. Overall, these blood samples were taken from volunteers aged 18 years old and over who had read the patient information form and signed consent forms. The sera obtained from these 200 non-haemolysed human blood samples were transferred into Eppendorf tubes and stored at −20 °C until ELISA and MAT were performed.

### Analysis of Human Samples with ELISA

NovaLisa^®^ ELISA kits (NovaLisa/NovaTec Immundiagnostica GmbH, Germany) were used to test for *Leptospira* IgG antibodies in both the risk and control groups. Testing was performed in accordance with the manufacturer’s recommendations.

### Microscopic Agglutination Tests Analysis

The MAT analyses were carried out at the Etlik Veterinary Control and Research Institute in Ankara. This study used standard strains grown in an Ellinghausen-McCullough-Johnson-Harris medium. A Thoma slide was used to ensure that the live *Leptospira* count was set to 108/mL. The human and animal sera were diluted with phosphate-buffered saline (PBS) at ratios of 1:25 and 1:50, respectively. Equal amounts of each antigen (standard strains) were added to the diluted sera. Positive and negative sera were used as controls. Microplates were shaken gently and incubated at 30 °C (±1 °C) for 2 h–4 h. Next, 5 μL of the serum–antigen mixtures were taken from the microplates using an automatic micropipette, put on slides and observed with a 10× objective dark field microscope. In the 200 human sera samples, antibodies against eight different serogroups of the *L. kirschneri* and *L. biflexa* species were studied. In the 289 bovine sera samples, antibodies against six different serogroups of the *L. kirschneri* and *L. interrogans* species were studied. The World Organisation for Animal Health (WOAH) and the Turkish Ministry of Health both consider 1:100 and 1:200 and above agglutinations positive for animals and humans, respectively (14, 15). Although we considered these values, some studies have evaluated bovine sera with a titer of 100 or greater against any *Leptospira* serovar positive (16).[Table t1-11mjms3105_oa]

### DNA Extraction and Real-Time Polymerase Chain Reaction Assay

Twenty milligram samples of kidney tissue were placed into 2 mL of sterile gasketed screw-cap beaded tubes. PBS was added to these, and the Indicaln Bioscience IndiMag Pathogen Kit was used for readymade tissue extraction. In the extraction, 200 μL tissue samples were used. The samples were homogenised in a Magna Lyser device at 7,043 × g for 60 s after being placed in ceramic bead tubes. The homogenized samples were spun at 11,001 × g/min for 3 min. Next, 200 μL of the supernatant was extracted for DNA extraction. The DNA samples, extracted using the Indical Bioscience Indimag automatic nucleic acid isolation device, were stored at −20 °C until PCR analysis.

Real-time PCR was applied by modifying Wu et al.’s (17) method. For the real-time PCR method, a LightCycler Taqman Master Kit (Roche Catalog No. 04535286001) and a Taqman probe that was fluorochrome marked at both the 5′ and 3′ ends were used. *Leptospira spp*. was detected in the amplification performed with a real-time PCR device (Roche LigshtCycler 480, Germany). *LipL32* F/R primers that target repetitive parts of the genome and a Taqman probe specific to the primers were used. The DNA of the *L. interrogans* serovar Pomona strain extracted at our institute was used as a positive control and ultra-distilled water was used as a negative control. In the first stage of the real-time PCR process for the *LipL32* gene, the DNA in the environment was denatured at 95 °C for 10 min and became single-stranded. The denaturation step of the amplification process was then completed by keeping the single-stranded DNA at 95 °C for 15 s. The results were evaluated using the qualitative detection programme of the real-time PCR device. Table 2 shows the primer we used and Table 3 shows the composition of the reaction mixture used in the real-time PCR procedure.

### Statistical Analysis

The patient information and laboratory results were entered into Microsoft Excel for Mac (version 16.70) to create tables and graphs. To calculate the number of cattle to be included in the study, descriptive analyses, such as the mean values and standard deviations of the ages of the cases, were carried out using the program’s analysis tools. The relationship between the variables was investigated using the chi-squared (χ^2^) test in the SPSS version 25.0 software and *P*-values less than 0.05 were considered significant.

## Results

In this study, samples taken from 289 cattle aged 1 year old–23 years old, from 100 professionals aged 19 years old–80 years old with a risk of leptospirosis and from 100 people aged 18 years old–82 years old without a risk of leptospirosis were investigated for the presence of *Leptospira* species. The characteristics of the cattle and the people included in the study are provided in Table 4.

Table 5 shows the PCR and MAT results from the kidney and serum samples of cattle fed in Erzurum and its districts as well as the ELISA results from human sera. PCR positivity was detected in only two cattle (in the fall semester). The Yates-confirmed χ^2^ test showed a low statistically significant increase in MAT positivity in favour of the fall semester based on the sample collection periods (χ^2^ = 3.5, df = 1, *P* = 0.061). The study revealed that 4.9% of rural cattle and 3.9% of urban cattle had positive results from the MAT analysis of their blood samples. The Yates-confirmed χ^2^ test showed no significant difference in antibody positivity between rural and urban cattle (χ^2^ = 0.015, df = 1, *P* = 0.903).

Table 6 shows the gender distribution of *Leptospira* IgG antibody positivity using ELISA in the risk and control groups. The study found no significant difference in *Leptospira* IgG antibody positivity between the risk group (14/100) and the control cases (18/100) (χ^2^ = 0.595, df = 1, *P* = 0.440).

Antibody positivity specific to five different *Leptospira* species at various titers was detected in the cattle. Table 7 lists the *Leptospira* species found in the humans by MAT. *Leptospira* antibody positivity was detected in two farmers and two control group cases.

## Discussion

Leptospirosis is a disease that is common primarily in tropical and subtropical regions with rainy and hot climates. In this study, the first information concerning *Leptospira* serogroups and epidemiology in cattle and humans was obtained in Erzurum, which is located at an altitude of approximately 2,000 m and has a rainy, cold and harsh continental climate. In our study, real-time PCR was used in the molecular diagnosis and MAT, which is accepted by WOAH as the gold standard, in the serological diagnosis of *Leptospira* in cattle. In this study, although PCR was found to be positive in two MAT-negative cattle, no positive results were obtained with PCR in any of the MAT-positive cases. These results show that 13 of the cattle were in the immune period, two were in the active infection period and 15 (5.2%) had encountered *Leptospira*. Five *Leptospira* serovars were commonly found in cattle in our region. These were Hardjo, Bratislava, Pomona and Icterohaemorrhagiae of *L. interrogans* and Dadas of *L. kirschneri*. People were found to have the serovar Hebdomadis and Icterohaemorrhagiae strains of *L. interrogans* and the serovar Dadas strain of *L. kirschneri*.

Numerous studies have utilised MAT analysis to detect the global prevalence of bovine leptospirosis. In Italy, an outbreak of *L. interrogans* serogroup Pomona detected using MAT was reported on cattle farms in Nebrodi Park (18). In cattle, MAT positivity has been reported at 73.0% in New Zealand (19), 27.8% in Uganda (20), 14.2% in Malaysia (21) and 25.8% in Iran (22). In Turkey, it was found to be 3.6% in the Thrace region (23) and in some provinces of Eastern Anatolia, 17.8% seropositivity was reported (24). As can be seen from the data above, the seroprevalence of bovine leptospirosis varies by country and region, and the rate in our region (5.2%) is below the world average.

*L. interrogans* serovar Hardjo (serogroup Sejroe) is most common in cattle, according to research conducted in several countries, including ours (23, 25–27). The presence of this serovar in cattle urine and genital tract discharge suggests environmental spread, and it is common in Malaysia, Argentina, Chile, India and Europe (25, 28). In this study, it was found to be the most common serogroup in Erzurum. We found antibodies that fight against the Bratislava, Pomona and Icterohaemorrhagiae serovars of *L. interrogans* and the Dadas serovar of *L. kirschneri*. Pasture farming, contaminated animals, other infections and horse-cattle cohabitation affect Erzurum cattle leptospirosis positivity. Dogs on Erzurum cattle farms may spread leptospirosis. Older animals that have been exposed to *Leptospira* may infect younger animals. Rural wild pigs may spread Pomona serovar to herds in pastures and the water sources where cattle drink. Red foxes, which prey on rodents in our region, can contaminate cattle and humans if relocated.

The bovine kidneys of two elderly animals tested positive using real-time PCR but negative for MAT. This indicates that the two strains may be from different strains not included in the standard panel. A comparable outcome was achieved in research carried out in Lebanon (29). The study found that the correlation between bovine blood PCR results and MAT analysis was poor. A Brazilian study found that 50% of the animals’ urine samples that had tested positive using PCR were negative when analysed using MAT (30). MAT standard panels differ among countries because of the varying prevalence of *Leptospira* serovars, usually consisting of five to seven serovars (25).

Numerous studies have examined the molecular prevalence of *Leptospira* in farm animals. In Brazil, 5.8% of cattle kidney samples and 14.9% of their urine samples carried the bacteria (26), 21% in New Zealand (21), and 7.2% in the US (31). Turkey has conducted few *Leptospira* molecular studies. A PCR study on cattle urine in Diyarbakır, Elazığ and Malatya provinces revealed 4.0% positivity (12). Immunohistochemical testing had a 1.0% positivity rate in Isparta, 9.4% in Diyarbakır and 24.2% in Erzurum in aborted foetuses (11, 13, 32). The literature shows that *Leptospira* DNA positivity rates vary widely by country and region. It is encouraging that our region and its surroundings have a lower positivity rate. The presence of bacteria as the active infection agent suggests that *Leptospira* infections should be monitored. Our study found that rural and urban farm animals had a similar risk of leptospirosis. Rural cattle may have higher MAT positivity than urban cattle due to stagnant water or contaminated pastures.

In Egypt, seropositivity has been reported in 3.6% of cattle and 6.2% of humans using ELISA (33). Leptospirosis seropositivity in humans has been reported at 4.0% in the US Virgin Islands (34) and at 12% in slaughterhouse workers in Çorum Province, Türkiye (35). In our study, farmers had the highest *Leptospira* IgG antibody positivity rates, followed by butchers and veterinarians. The risk group and control group cases did not exhibit significant differences in *Leptospira* antibody positivity when compared. In the control group, positivity was detected in only two cases using the MAT test, which is considered the gold standard in diagnosis and a negative result was obtained using ELISA in one of these cases.

## Conclusion

The general population is as susceptible to *Leptospira* as farmers, butchers and veterinarians. Many factors spread *Leptospira* bacteria in Erzurum’s environment. Many wild and domestic animals that roam freely in nature pose the most important risk. There is no technical and scientific protocol for the epidemiological surveillance, clinical management, prevention or control of this infection in Erzurum and its region. Therefore, we need to emphasise that leptospirosis is a disease that should be monitored carefully. People who touch infected animals or encounter suspicious situations should contact the hospital. Municipal services, such as environmental cleaning and infrastructure, must be combined with health institutions’ public awareness campaigns about leptospirosis infection, transmission and prevention. Animal, human and environmental health professionals must work together to control leptospirosis and create control plans. In conclusion, a prospective leptospirosis study was conducted for the first time in our region using real-time PCR, MAT, and ELISA on cattle and human serum and on kidney samples. Unfortunately, leptospirosis epidemiology research in Türkiye is scarce. We believe that this prospective study could significantly address this gap.

## Figures and Tables

**Table 1 t1-11mjms3105_oa:** Antigen panel used in MAT analysis in cattle and humans

Species	Serovar	Strain	In cattle or human
*L. kirschneri*	Dadas	Dadas I	Both of them
*L. interrogans*	Bratislava	Jez Bratislava	Both of them
*L. interrogans*	Canicola	Hond Utrecht IV	Both of them
*L. interrogans*	Hardjo	Hardjoprajitno	Both of them
*L. interrogans*	Pomona	Pomona	Both of them
*L. interrogans*	Icterohaemorrhagiae	RGA	Cattle only
*L. interrogans*	Icterohaemorrhagiae	Weijinberg	Humans only
*L. interrogans*	Hebdomadis	Hebdomadis	Humans only
*L. biflexa*	Patoc	Patoc I	Humans only

**Table 2:** Real-time PCR primer-probes

Primer-probes (17)
*LipL32*F 5′-GGATCCGTGTAGAAAGAATGTCGG-3′
*LipL32*R 5′-GTCACCATCATCATCATCGTC C-3′
Prob-FAM-5′-ATGCCTGACCAAATCGCCAAAGCTGCGAAA-TAMRA-3′

**Table 3:** Content of the reaction mixture used in real-time PCR

Content of the reaction mixture	Amount (μL)
H_2_O (nuclease-free water)	9
*LipL32*-forward primer, 10 μM	0.5
*LipL32*-reverse primer, 10 μM	0.5
FastStart mix	4
Taqman probe, 4 μM	1
Sample DNA	5

Total	20

**Table 4:** Cases characteristics

Characteristics	Data
Cattle (*n* = 289)	
Male/female	57/232
Age, Mean ± SD; (range)	6.53 ± 4.4 (1–23)
Farm settlement looked after	
Rural	162
Urban	127
People (risk group, *n* = 100)	
Male/female	84/16
Age, Mean ± SD; (range)	43.23 ± 13.16 (19–80)
Occupation:	
Farmer	58
Veterinarian	25
Butcher	17
People (control group, *n* = 100)	
Male/female	43/57
Age, Mean ± SD; (range)	45.35 ± 17.19 (18–82)

**Table 5:** PCR and MAT results in cattle

Parameters	Positive *n* (%)	Negative *n* (%)	*P*-value
Assay in cattle			
PCR (in kidney samples, *n* = 289)	2 (0.7)	287 (99.3)	–
MAT (in sera samples, *n* = 289)	13 (4.5)	276 (95.5)	
MAT results by period			
Spring period (*n* = 151)	3 (2.0)	148 (98)	0.0614
Autumn period (*n* = 138)	10 (7.2)	128 (92.8)	
MAT results by residential district			
Rural (*n* = 162)	8 (4.9)	154 (95.1)	0.9030
Urban (*n* = 127)	5 (3.9)	122 (96.1)	

**Table 6:** ELISA-*leptospira* IgG antibody positivity in risk groups and controls

Groups	Positive *n* (%)	Negative *n* (%)
Farmer (*n* = 58)	10 (17.2)	48 (82.8)
Veterinarian (*n* = 25)	2 (8.0)	23 (92.0)
Butcher (*n* = 17)	2 (11.8)	15 (88.2)
Controls (*n* = 100)	18 (18.0)	82 (82.0)

**Table 7:** *Leptospira* serovars identified by MAT assay in cattle and humans

Serovars	*n* (%)
In cattle	
*L. interrogans* serovar Hardjo	6 (46.2)
*L. kirschneri* serovar Dadas	3 (23.1)
*L. interrogans* serovar Bratislava	2 (15.4)
*L. interrogans* serovar Pomona	1 (7.7)
*L. interrogans* serovar Icterohaemorrhagiae	1 (7.7)
Total	13 (100)
In humans	
Risk group	
*L. interrogans* serovar Hebdomadis	1 (1.0)
*L. interrogans* serovar Icterohaemorrhagiae	1 (1.0)
Control	
*L. interrogans* serovar Icterohaemorrhagiae	1 (1.0)
*L. kirschneri* serovar Dadas	1 (1.0)
